# Supplementary material for: Effects of Electrocardiographic Monitoring Education on Nurses’ Confidence and Psychological Stress: An Online Cross-Sectional Survey in Japan
Source: Int J Environ Res Public Health. 2022 Apr 14;19(8):4742. doi: 10.3390/ijerph19084742 (PMC9024973; doi:10.3390/ijerph19084742)
Supplement: Supplementary file 1 [file ijerph-19-04742-s001.zip › ijerph-1595669-supplementary.pdf]

**QUESTIONNAIRE FOR NURSES**

Gender: Female / Male

Nursing licence type: State registered nurse / State enrolled nurse

Your post-graduate years: 1-5 / 6-10 / Over 11

Hospital scale in your medical facility (beds): Under 99 / 100-299 / Over 300

Night shift

Manager position

Department: Internal Medicine / Surgery / Paediatrics / Obstetrics and Gynaecology / Emergency room / Intensive care units or High care units / Dermatology / Ophthalmology / Otolaryngology / Urology / Rehabilitation / Psychiatry / Gastroenterology / Cardiology / Nephrology / Diabetes and endocrinology / Rheumatology and clinical immunology / Allergy / Haematology / Neurology / Psychosomatic medicine / Infectious disease medicine / Oncology / Thoracic surgery / Cardiovascular surgery / Respiratory surgery / Breast surgery / Thyroid surgery / Paediatric surgery / Proctology / Orthopaedic surgery / Neurosurgery / Plastic surgery

1. Did you receive pre-graduate ECG monitoring education?
   Yes / No
2. Did you receive post-graduate ECG monitoring education?
   Yes / No
3. How useful do you think pre-graduate ECG monitoring education is?
   Not useful / Minimally useful / Modestly useful / Useful
4. How useful do you think post-graduate ECG monitoring education is?
   Not useful / Minimally useful / Modestly useful / Useful
5. Did you have helpful experience with ECG monitoring?
   1-4 times / Over 5 times
6. Do you have confidence regarding ECG monitoring?
   Not confident at all / Not very confident / Little confident / Confident
7. Do you have psychological stress regarding ECG monitoring?
   Very stressful / Slightly stressful / Not very stressful / Not stressful at all
